# Immunoenhancing Effects of *Euglena gracilis* on a Cyclophosphamide-Induced Immunosuppressive Mouse Model

**DOI:** 10.4014/jmb.2112.12035

**Published:** 2021-12-29

**Authors:** Hyeonji Yang, Kwanyong Choi, Kyeong Jin Kim, Soo-yeon Park, Jin-Young Jeon, Byung-Gon Kim, Ji Yeon Kim

**Affiliations:** 1 Department of Food Science and Technology, Seoul National University of Science and Technology, Seoul 01811, Republic of Korea; 2 Department of Nano Bio Engineering, Seoul National University of Science and Technology, Seoul 01811, Republic of Korea; 3 Lab of Nanobio, Seoul National University of Science and Technology, Seoul 08826, Republic of Korea; 4 BIO R&D center, Daesang Corp., Icheon 17384, Republic of Korea

**Keywords:** Immune enhancement, cyclophosphamide, *Euglena gracilis*, β-glucan, splenocytes, dectin-1

## Abstract

In this study, the effects of the immune stimulator *Euglena gracilis* (Euglena) in cyclophosphamide (CCP)-induced immunocompromised mice were assessed. The key component β-1,3-glucan (paramylon) constitutes 50% of *E. gracilis*. Mice were orally administered *Euglena* powder (250 and 500 mg/kg body weight (B.W.)) or β-glucan powder (250 mg/kg B.W.) for 19 days. In a preliminary immunology experiment, ICR mice were intraperitoneally injected with 80 mg of CCP/kg B.W. during the final 3 consecutive days. In the main experiment, BALB/c mice were treated with CCP for the final 5 days. To evaluate the enhancing effects of *Euglena* on the immune system, mouse B.W., the spleen index, natural killer (NK) cell activity and mRNA expression in splenocytes lungs and livers were determined. To detect cytokine and receptor expression, splenocytes were treated with 5 µg/ml concanavalin A or 1 µg/ml lipopolysaccharide. The B.W. and spleen index were significantly increased and NK cell activity was slightly enhanced in all the experimental groups compared to the CCP group. In splenocytes, the gene expression levels of tumor necrosis factor-α, interferon-γ, interleukin (IL)-10, IL-6, and IL-12 receptor were increased in the *E. gracilis* and β-glucan groups compared to the CCP group, but there was no significant difference. Treatment with 500mg of *Euglena*/kg B.W. significantly upregulated dectin-1 mRNA expression in the lung and liver compared to the CCP group. These results suggest that *Euglena* may enhance the immune system by strengthening innate immunity through immunosuppression.

## Introduction

Immunity is the ability to resist harmful microorganisms with specific and nonspecific responses. The immune system plays a defensive role, preventing pathogens from entering the body. The immune system is based on complex and sophisticated biological mechanisms in immune cells, such as dendritic cells (DCs), macrophages, and lymphocytes [1]. These cells play the main roles in immune responses: DCs process antigen material and present it on the cell surface to stimulate T cells [2]; macrophages play a critical role in the nonspecific defense response, facilitating the initiation of specific defense mechanisms by recruiting other immune cells [3, 4], and lymphocytes are white blood cells in the vertebrate immune system [1]. The immune system protects a host against infectious agents and is commonly categorized into innate and adaptive immunity [5]. Innate immunity plays a role in the nonspecific defense against pathogens through initial inflammatory responses or phagocytosis [6]. Adaptive immunity is specific and triggers immunological memory after initial immune responses are induced. All these immune responses are modulated by cytokines, which are involved in signaling between proximal and/or distant cells [7].

*Euglena gracilis* (*Euglena*) is a spindle-shaped microalgae belonging to the Euglenaceae family and has features of both plants and animals. *Euglena* has a nucleus, an eyespot, a contractile vacuole, a flagellum, and chloroplasts with pigments [8-10]. *Euglena* is attracting attention as a raw material for use in new functional foods [11-13]. *Euglena* contains a wide range of nutrients, including minerals, amino acids, vitamins, and fatty acids, and it is used in food and/or nutritional supplements [10, 14]. *Euglena* mainly consists of β-1,3-glucan (paramylon), which enhances the immune system by inducing cytokine secretion, natural killer (NK) cell activity, and dectin-1 expression [14, 15]. Paramylon, also known as β-glucan, is an insoluble dietary fiber [14]. Various functions of *Euglena* or β-glucan extracted from *Euglena gracilis* have been demonstrated in previous studies. *Euglena* intake attenuated hyperglycemia and liver fat accumulation [16]. The anti-inflammatory and visceral fat accumulation inhibition effects of *Euglena* have been demonstrated in vivo [17, 18]. Another animal study has shown that paramylon fiber prevents obesity [19]. In a human study, *Euglena* enhanced the immune response in the mucosal surface and showed potential as a functional food [20].

Cyclophosphamide (CCP) has been used extensively in the clinic as an alkylation agent [21]. CCP inhibits cancer cell proliferation but damages lymphoid organs, particularly the spleen, and decreases the number of lymphocytes, leading to immunosuppression [22, 23].

Although some previous studies have demonstrated immunomodulatory effects of *Euglena gracilis* and β-glucan, to the best of our knowledge, few studies have demonstrated the effects of *Euglena gracilis* powder against CCP-induced immunosuppression in vivo. In the present study, the immunostimulatory effects of *Euglena* and β-glucan were investigated in a CCP-induced immunosuppressive mouse model. We sought to demonstrate the immune-enhancing potential of *Euglena* effects on CCP-induced immunosuppression in vivo, which are mediated through increased dectin-1 expression, NK cell activity, and immune-related cytokine expression. β-Glucan was the bioactive compound used in this study.

## Materials and Methods

### Materials

Roswell Park Memorial Institute (RPMI) 1640 medium was obtained from Gibco-Life Technologies (USA). Fetal bovine serum (FBS), penicillin–streptomycin mixture (Biowest, France) and Dulbecco’s phosphate buffered saline (DPBS) were purchased from Biowest (Nuaille’, France). Blood cell lysis buffer, amphotericin B, trypan blue, concanavalin A (ConA) and lipopolysaccharide (LPS) were obtained from Sigma–Aldrich (USA).

### Animals and Cell Culture

For the preliminary experiment, five-week-old male ICR mice were obtained from Hana Bio (Korea), and six-week-old male BALB/c mice from DooYeol Biotech (Korea) were used for the main experiment. The animals were maintained in pathogen-free, environmentally controlled rooms maintained at 20-26°C, with a relative humidity of 30-70% and a 12-h dark–light cycle for at least a week before the start of the experiment. They were fed a commercial diet and water. The experimental protocol for the preliminary study was approved by the Southeast Medi-Chem Institute (Receipt Number: SEMI-21-006), and the experimental protocol for the main study was approved by the Korea Radioisotope Center (Receipt Number: Kirams 2021-0023).

YAC-1 (KCLB No. 40160) cells were obtained from Korea Cell Line Bank (Korea). YAC-1 cells were cultured in RPMI 1640 medium containing 10% FBS and a 1% penicillin–streptomycin mixture at 37°C with 5% CO_2_.

### Preparation of the Sample

*Euglena* powder was provided by Daesang Corp., R&D Center (Korea). *Euglena gracilis* DSW1 cells (KCTC 13930BP) were grown in medium containing glucose, dibasic potassium phosphate, L-glutamic acid, DL-malic acid, and cyanocobalamin in a jar fermenter at 28°C for 6 days. After cultivation, the cells were harvested and washed with filtered water. Then, these cells were sterilized with High Temperature/Short Time (HTST) and dried. The composition of *Euglena* powder was analyzed by the Korea Health Supplement Institute (Korea)(Table 1).

*Euglena* powder contains 665.61 mg of β-glucans/g. These *Euglena*-derived β-glucans were analyzed by 1H NMR spectroscopy at the Korea Basic Science Institute (Korea) and found to be β-1,3-glucans.

The β-glucans were prepared as follows: *Euglena* DSW1 cells were grown as heterotrophs in the darkness. After cultivation, the cells were collected by centrifugation at 4,000 ×*g* for 10 min and washed twice with distilled water. The pH of the washed cells was adjusted with NaOH to pH 12.5 and extracted at 60°C for 1 h. The extract was separated by centrifugation at 4,000 ×*g* for 10 min, sterilized with HTST and dried. The paramylon used in this contained 97% β-glucan.

### Immunosuppressive Mouse Model and Treatment

**Fig. 1:**
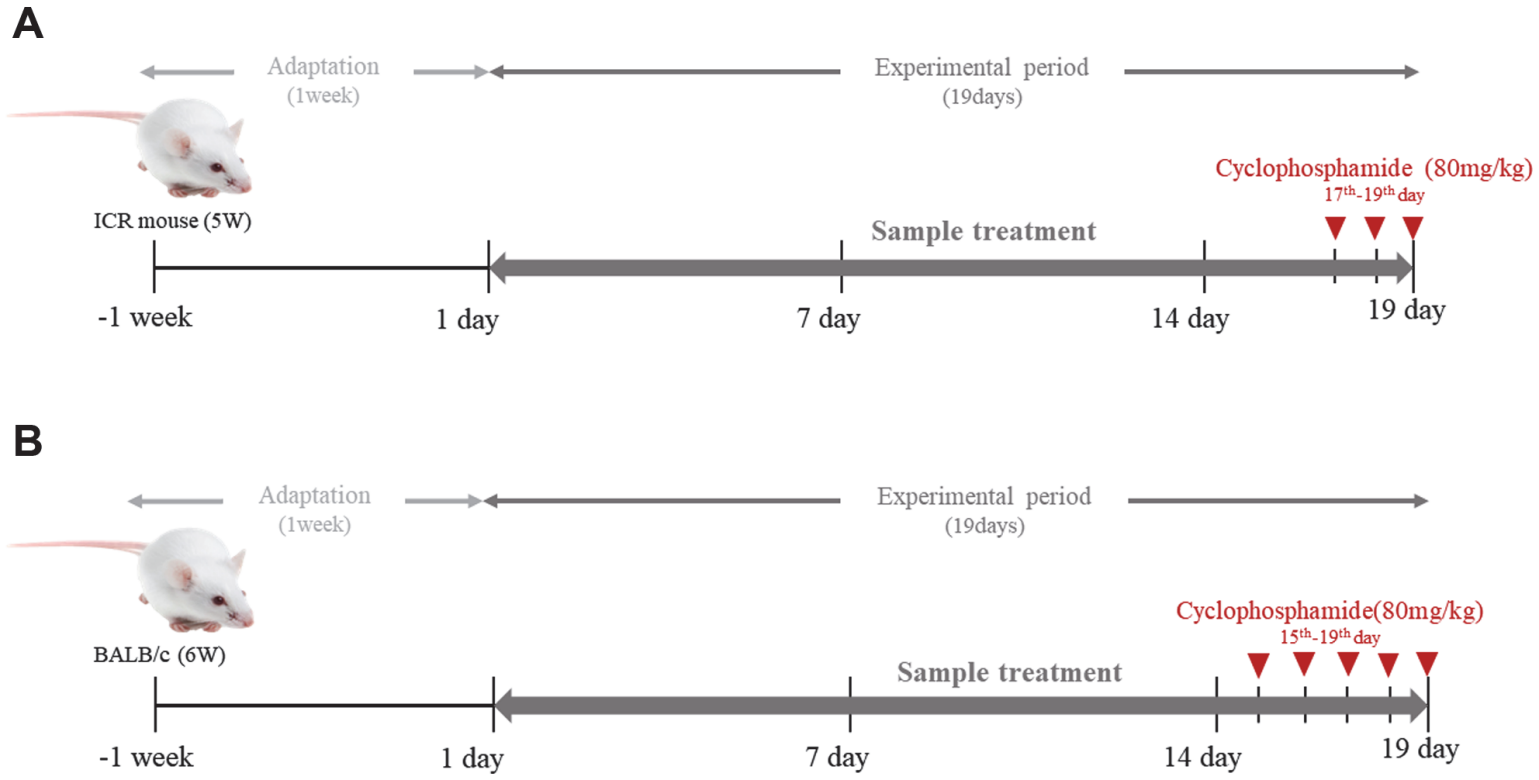
Animal experimental procedure. After an adaptation period of one week, *Euglena gracilis* or β-glucan was orally administered every day except in the CON and CCP groups for 19 days. Cyclophosphamide (CCP) was administered to ICR mice intraperitoneally, except to those in the CON group, for the final 3 consecutive days to ICR mice (**A**) and the final 5 consecutive days to BALB/c mice, except to those in the CON group (**B**).

After 1 week of adaptation, ICR and BALB/c mice were randomly divided into five groups. In the preliminary experiment, ICR mice were assigned to each of the following groups (9 mice per group): the normal control (CON), cyclophosphamide-only (CCP), low concentration *Euglena* (LE, 250 mg/kg B.W.), high concentration *Euglena* (HE, 500 mg/kg B.W.), and β-glucan (BG; 250 mg/kg B.W.) groups. During the final 3 days of the experiment, the mice in all groups except those in the CON group were intraperitoneally injected with 80 mg of CCP/kg B.W. In the main experiment, BALB/c mice were allocated to five groups identical to those comprising ICR mice, but the number of BALB/c mice in each group differed to obtain enough splenocytes: In the CON group, *n* = 10; in the CCP group, *n* = 20; in the LE group, *n* = 20; in the HE group; n =20, and in the BG group, *n* = 20. The mice in the CON and CCP groups were orally administered saline. The mice in the *Euglena*-treated groups were administered *Euglena* orally at concentrations of 250 mg/kg B.W. and 500 mg/kg B.W., and the mice in the β-glucan group were orally administered β-glucan at a concentration of 250 mg/kg B.W. for 19 days. In the final 5 days, all mice except those in the CON group were treated with CCP. The ICR and BALB/c mice in the respective CON groups were injected with saline. On day 19, the mice were fasted for 12 h and sacrificed. The spleens, lungs, and livers were extracted. The animal experimental procedure is shown by Fig. 1.

### Isolation of Splenocytes

The spleens obtained from ICR and BALB/c mice were washed with sterilized saline and weighed, and the following index was calculated: spleen index (mg/g) = spleen weight (mg)/B.W. (g). Spleens were stored in cold RPMI 1640 before splenocyte extraction. The spleens were gently pressed through a syringe and filtered through a 70-μm cell strainer (SPL Life Sciences, Korea). Red blood cells were removed by treatment with red blood cell lysis buffer and washed with RPMI 1640 medium.

### Cell Culture for Measuring Cytokine Expression

The splenocytes were incubated in RPMI 1640 medium containing 10% FBS, a 1% penicillin–streptomycin mixture, and 2.5 μg/ml amphotericin B. Splenocytes isolated from BALB/c mice were seeded in 12-well plates at 1 × 10^6^ cells/well and incubated with 5 μg/ml ConA or 1 μg/ml LPS for 24 h at 37°C with 5% CO_2_.

### Detection of LDH Cytotoxicity for Measuring the Natural Killer Cell Activity of Splenocytes

The activity of NK cell among splenocytes obtained from ICR mice was analyzed. YAC-1 cells were seeded at 1 × 10^4^ cells/well, and splenocytes (effector) were cocultured with YAC-1 (target) cells in 96-well plates as an effector cell:target cell ratio of 2:1. After 4 hours of incubation at 37°C in 5% CO_2_, the plates were centrifuged at 250 ×*g* for 10 min. 50 μl of supernatant was transferred into a clear 96-well microplate. LDH activity was determined using a CytoTox 96 non-radioactive cytotoxicity assay (Promega Corp., USA). CytoTox 96 reagent was added to each supernatant. The absorbance of each well was measured at 490 nm. NK cell activity was calculated using the following equation: cytotoxicity (%) = {(experimental - effector spontaneous - target spontaneous)/(target maximum - target spontaneous)} × 100

### RNA Extraction from Tissues and Cells and Analysis of Immune Gene Expression with Quantitative RT–PCR

Total RNA was extracted from livers, lungs and splenocytes treated with ConA or LPS using TRIzol reagent (Life Technologies, USA) following the manufacturer’s protocol. Then, total RNA was reverse-transcribed using a cDNA reverse transcription kit (Roche, Switzerland). The Universal Probe Library (UPL) method was used to quantify the expression of interferon-γ (IFN-γ), tumor necrosis factor-α (TNF-α), interleukin (IL)-10, IL-6, and IL-12 receptor β1 in splenocytes and dectin-1 in livers and lungs using a Light Cycler 96 system (Hoffmann La Roche). Glyceraldehyde 3-phosphate dehydrogenase (GAPDH) gene expression was used for normalization to calibrate the gene expression tool. The relative mRNA expression, normalized to that of GAPDH, was calculated using the comparative CT method. The sequences of the sense and antisense primers are presented in Table 2.

### Statistical Analysis

All experimental results are presented as the means ± standard error (SE). Mean differences were analyzed by one-way analysis of variance (ANOVA) and Duncan’s multiple range tests with SAS software (version 9.4; SAS Institute Inc., USA). A *p* value < 0.05 was considered to be statistically significant.

## Results

### Effects of *Euglena* on B.W. and the Spleen Index

**Fig. 2:**
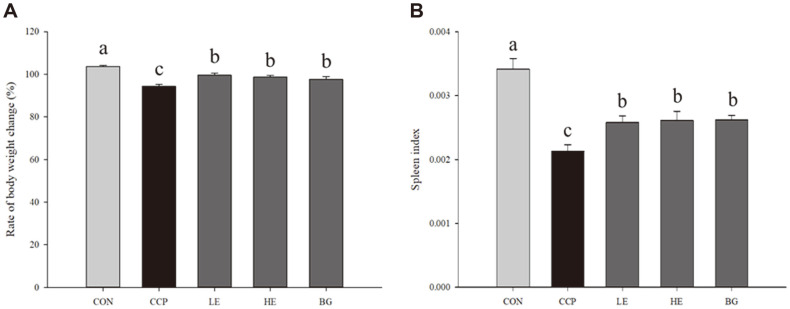
Effects of *Euglena* and β-glucan on the body weight (B.W.) and spleen index in ICR mice. CON, normal control; CCP, cyclophosphamide-only; LE, CCP+250 mg of *Euglena gracilis*/kg B.W.; HE, CCP+500 mg of *Euglena gracilis*/kg B.W.; BG, CCP+250 mg of β-glucan /kg B.W. (**A**) B.W. change rate after CCP injection. Ratio of B.W. on day 19 compared to that on day 17. (**B**) Spleens in all groups were weighed, and the spleen index was calculated. Data with different letters within a row are significantly different at *p* < 0.05, as determined by Duncan’s multiple range test. Values from small to large are arranged in alphabetical order.

To investigate the protective effect of CCP on immune suppression in ICR mice, the change in weight gain rate was measured and compared with that with the initial CCP administration. B.W. on day 17 was considered to be the baseline and was calculated as follows: B.W. change ratio (%) = B.W. on day 19 (g)/B.W. on day 17 (g) × 100. As shown in Fig. 2A, the B.W. of the mice in CCP-treated groups (the CCP, LE, HE, and BG groups) was decreased compared with that of the CON group (103.72 ± 0.51%). The B.W. loss ratio of the CCP group was markedly decreased (94.42 ± 0.88%) compared to that of the CON group (*p* < 0.05). Weight loss due to CCP treatment was significantly attenuated in mice administered *Euglena* or β-glucan (LE group, 99.61 ± 0.78%; HE group, 98.73 ± 0.84%; and BG group, 97.69 ± 1.25%; *p* < 0.05).

As shown in Fig. 2B, the spleen index of the CCP group was significantly lower (0.0213 ± 0.0010) than that of the CON group (*p* < 0.05). The LE, HE, and BG groups showed significant increases (LE group, 0.0258 ± 0.0010; HE group, 0.0261 ± 0.0014; and BG group, 0.0262 ± 0.0007) compared with the CCP group (*p* < 0.05), but these differences did not lead to recovery as much as level of CON group.

### Effects of *Euglena* on Natural Killer Cell Activity

**Fig. 3:**
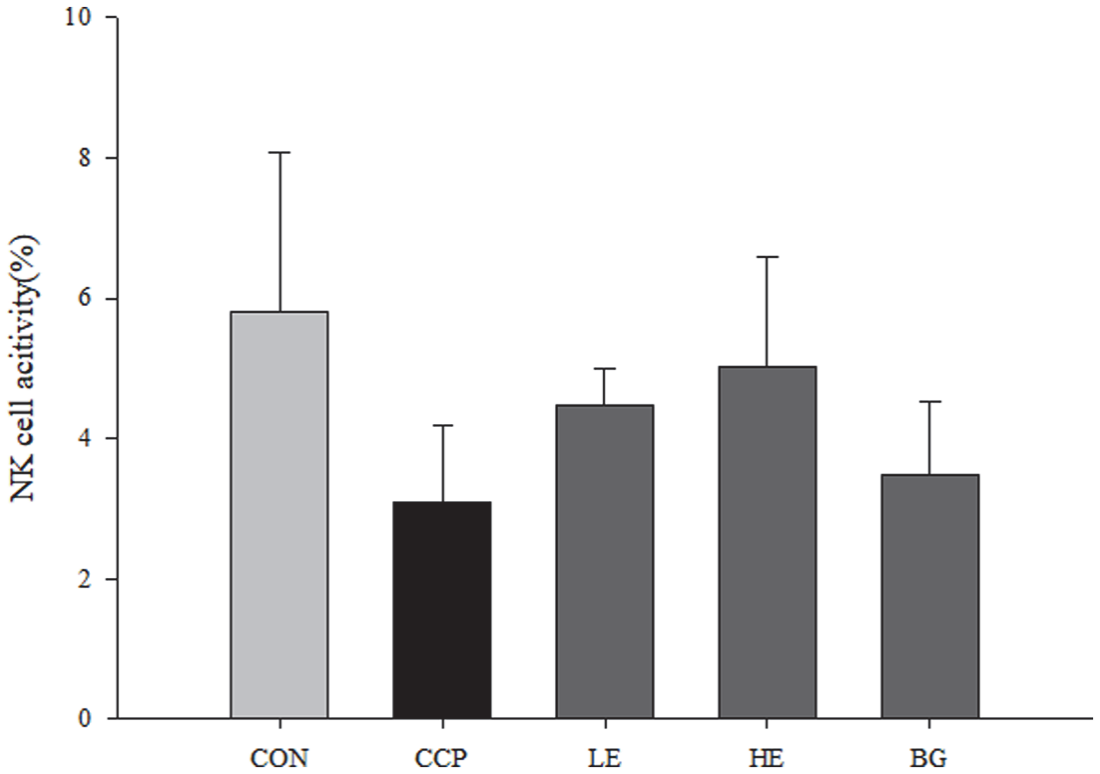
Effects of *Euglena* and β-glucan on natural killer (NK) cell activity in ICR mice. Splenocytes were cocultured with YAC-1 cells for 4 hours. The cytotoxicity of the splenic NK cells was measured. Data with different letters within a row are significantly different at *p* < 0.05, as determined by Duncan’s multiple range test.

To determine NK cell activity, the relative amount of lactate dehydrogenase (LDH) in the supernatant obtained from cocultured splenocytes and YAC-1 cells was assayed. The LDH levels between the groups (CON, CCP, LE, HE, and BG groups) did not differ. Specifically, in the CCP group, the LDH level was decreased (3.097 ± 1.080%) compared to that in the CON group. In the treatment groups (LE, HE, and BG groups), the LDH level was slightly upregulated (LE group, 4.478 ± 0.518%; HE group, 5.015 ± 1.567%; and BG group, 3.494 ± 1.036%) compared with that in the CCP group. Therefore, the NK cell activity level in the HE group was the highest among experimental groups (Fig. 3).

### Effects of *Euglena* on the mRNA Expression of Cytokines and a Cytokine Receptor

**Fig. 4:**
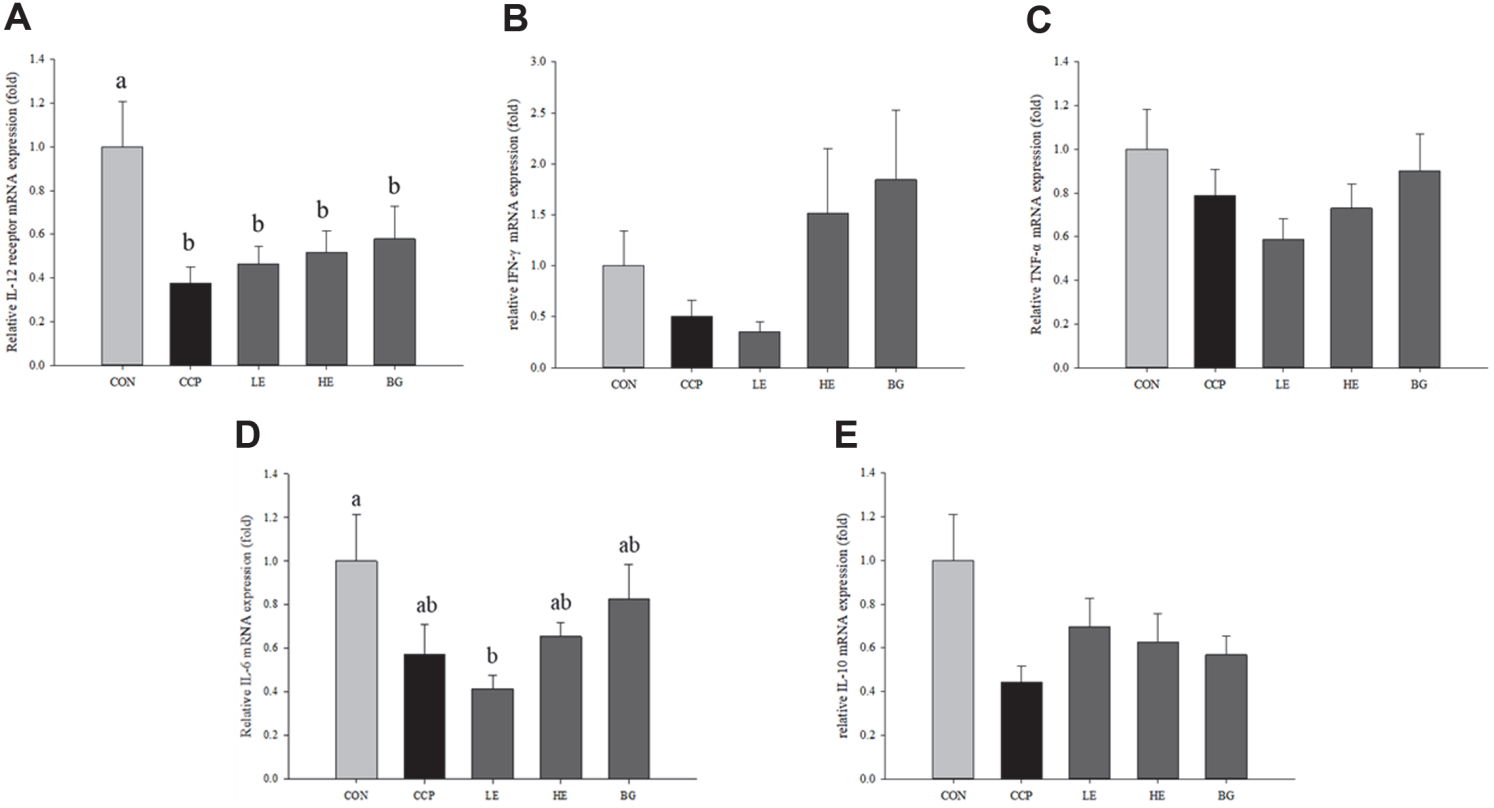
Effects of *Euglena* and β-glucan on the relative gene expression of cytokines involved in the immune system in BALB/c mice. Splenocytes were isolated and stimulated with 1 μg/ml lipopolysaccharide (LPS) or 5 μg/ml concanavalin A (ConA), which are mitogens. After 24 hours, total RNA was extracted from cells. Cytokine expression was analyzed in LPS-stimulated cells, (**A**) IL-12Rβ1, and in ConA-stimulated cells, (**B**) IFN-γ, (**C**) TNF-α, (**D**) IL-6, and (**E**) IL-10. Data with different letters within a row are significantly different at *p* < 0.05, as determined by Duncan’s multiple range test. Values from small to large are arranged in alphabetical order.

Splenocytes were isolated from BALB/c mouse spleens, and then, the relative mRNA expression of IL-12Rβ1 in the LPS-treated cells was investigated. TNF-α, IFN-γ, IL-6, and IL-10 expression levels in the ConA-treated cells were assayed by qRT–PCR. The mRNA expression of IL-12Rβ1 was significantly decreased in the CCP group (0.377 ± 0.073-fold) compared to the CON group (*p* < 0.05). The levels in the treatment groups (LE, HE, and BG groups) was gradually increased (0.466 ± 0.078-fold) compared to that in the CCP group, but there was no significant difference (Fig. 4A).

As shown in Fig. 4B, IFN-γ mRNA expression levels in the HE and BG groups tended to increase (HE, 1.520 ± 0.632-fold and BG, 1.845 ± 0.686-fold) to a greater level than the CON group, although there were no significant differences. The expression of proinflammatory cytokines, such as TNF-α and IL-6, showed similar tendencies. At dose of 250 mg/kg B.W. (LE group), mRNA expression of TNF-α and IL-6 was reduced (TNF-α, 0.588 ± 0.094-fold and IL-6, 0.413 ± 0.062-fold) in comparison to the CCP group (TNF-α, 0.790 ± 0.119-fold and IL-6, 0.0572 ± 0.135-fold). However, administration of β-glucan slightly increased the levels of TNF-α (0.903 ± 0.166-fold) and IL-6 (0.825 ± 0.161-fold) compared to that in the CCP group. Although the difference was not significant for the HE group, TNF-α mRNA expression was slightly reduced (0.731 ± 0.111-fold), whereas IL-6 mRNA expression was slightly increased (0.654 ± 0.063-fold). The results are presented in Fig. 4C and 4D. In the treatment groups (LE, HE, and BG), the mRNA expression of IL-10 it showed an upregulation tendency (LE group, 0.698 ± 0.129-fold; HE group, 0.626 ± 0.129-fold; and BG group, 0.569 ± 0.086- fold) compared to the CCP group, but it was not significantly different. In the CCP group, IL-10 expression tended to be downregulated (0.443 ± 0.074-fold) compared to that in the CON group (Fig. 4E).

### Effects of *Euglena* on the mRNA Expression of Dectin-1

**Fig. 5:**
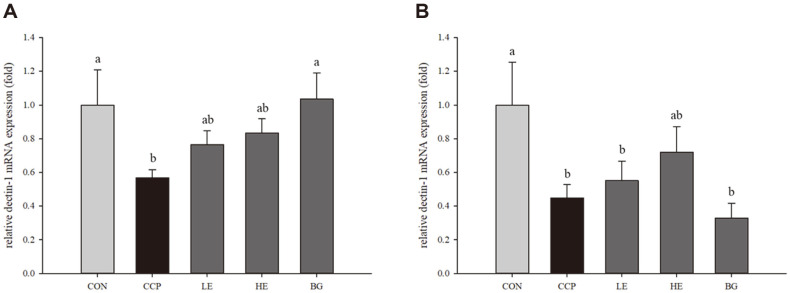
Effects of *Euglena* and β-glucan on the relative gene expression of dectin-1 in BALB/c mice. The lungs and livers were collected from all mice. Total RNA was extracted from the lungs and livers, and cDNA was reverse transcribed. The relative expression of dectin-1 was quantified using qRT–PCR. Dectin-1 mRNA expression was measured in the lungs (**A**) and livers (**B**). Data with different letters within a row are significantly different at *p* < 0.05, as determined by Duncan’s multiple range test. Values from small to large are arranged in alphabetical order.

The mRNA expression of dectin-1 in the CCP groups was significantly reduced (0.568 ± 0.0485-fold) in both lungs and livers compared to that in the CON group (*p* < 0.05). In the lungs, the dectin-1 levels in the LE and HE groups were noticeably increased (LE group, 0.7660 ± 0.0803-fold and HE group, 0.8347 ± 0.0832-fold) compared to the CCP group (0.568 ± 0.0485-fold). Moreover, notably, the level of dectin-1 in the BG group treated with 250 mg of β-glucan/kg B.W. was significantly increased (1.0347 ± 0.1546-fold) to the level of CON group (*p* < 0.05). The results are shown in Fig. 5A.

In the livers, decin-1 mRNA expression in the HE group was obviously increased (0.7193 ± 0.1519-fold) compared to that in the CCP group (*p* < 0.05). The level in the LE group was slightly increased compared with that in the CCP group, whereas there was a decreasing tendency in the BG group compared with the CCP group (Fig. 5B).

## Discussion

To the best of our knowledge, this is the first study to use an immunosuppressive mouse model treated with CCP to confirm the immunoenhancing effect of *Euglena*. CCP has been widely used to induce immunosuppression in vivo. In the present study, 80 mg of CCP/kg B.W. was intraperitoneally administered for 3 or 5 days, and immunosuppression was confirmed with the following results: The B.W., spleen index, and mRNA expression of the IL-12 receptor, IL-6, and dectin-1 were significantly reduced by injection with CCP (*p* < 0.05). This demonstrated that CCP strongly suppresses the immune system by reducing the number of leucocytes and the activity of lymphocytes [24-26]. Our study showed a significant increase in dectin-1 expression, in contrast to NK cell activity and cytokine expression after *Euglena* intake.

The spleen is an immune organ corresponding to the second lymphatic tissue [27, 28]. The spleen is involved in both immunity and hematopoiesis [29, 30], providing an immune response against mycobacterial infections [1]. The spleen is composed of immune cells, such as macrophages, monocytes, B and T cells, and NK cells, which have different immune functions, and modulate immune responses [31, 32]. The cells extracted from the spleen are called splenocytes. The spleen index was improved by the administration of *Euglena* and β-glucan. This result indicated that *Euglena* and β-glucan improved the immune system and hematopoietic process (Fig. 2B).

Lymphocytes are primarily composed of NK cells, T cells, and B cells. As part of the lymphocyte class, NK cells are best known for nonspecific killing of pathogens and infected cells, and it has been demonstrated that NK cells play a role in controlling infection in the earliest phases of the immune system [33, 34]. NK cells are in the primary line of defense that protects the body from invasion and infection by pathogens. When normal cells are infected with a virus, NK cells are rapidly activated to destroy both abnormal cells and virus-infected cells without presensitization to protect them [35-38]. Based on this information, we predicted that NK cells play an important role in the pathogen-induced immune system and the control of tumor growth [39, 40]. In addition, they also have been shown to modulate immune responses via the release of immune-regulating cytokines and chemokines [41, 42], modulation of DC activity, and granulocyte growth and differentiation [42-45]. In conclusion, NK cells are classified as innate immune cells with cytotoxicity against tumor cells and virus-infected cells, and also secrete several signaling substances such as IFN-γ, TNF-α, and chemokines. Through this action, NK cells play an important role in the innate immune responses, and play a role as a bridge from innate immune responses to adaptive immune responses [46, 47]. Increased NK cells activity indicates activation of cytotoxicity to tumors or infected cells, suggesting the potential to cause rapid innate immune responses from pathogens invading the body. In this investigation, NK cell activity was slightly increased in the *Euglena* and β-glucan treatment groups (Fig. 3). *Euglena* may have the potential to improve the immune system through the cytotoxicity induced by NK cells themselves and regulatory mechanisms. It is first time to study the effects of *Euglena* on NK cell activity, but in previous human studies, Lee *et al*. determined that β-1,3-glucan enhanced NK cell activity in peripheral blood mononuclear cells [48].

T lymphocytes are derived from bone marrow precursor cells and activated through maturation, selection and release in the thymus. Peripheral T cells are composed of various subsets depending on their function, such as naïve T cells, memory T cells, regulatory T cells, and helper T (Th) cells. The immune response by T cells is initiated when T cells encounter antigen and are stimulated by ligands presented by DCs [49]. Th cells, which are CD4+ T cells, play an important role in protecting the body from infection and secrete both proinflammatory cytokines (*e.g.*, IFN-γ, TNF-α, and IL-6) and anti-inflammatory cytokines (*e.g.*, IL-10). Th1 and Th2, Th9 and Th17 are Th cell [50] that help B cells by secreting diverse cytokines, which act as bridges between innate immunity and the adaptive immune response. B cells are derived from hematopoietic stem cells (HSCs) originating in bone marrow [51, 52]. Specifically, HSCs differentiate into common lymphoid progenitor cells and then develop into immature B cells in several stages [53]. Immature B cells move to the spleen from bone marrow and develop into mature B cells. B cells are keys to adaptive immunity because they secrete the immunoglobulins and cytokines necessary for humoral immunity and act as antigen-presenting cells [54]. T and B lymphocytes are regulated by cytokines secreted by immune cells such as macrophages, DCs, NK cells, and T and B cells. In the present study, the mRNA expression of cytokines from T and B cells was evaluated. *Euglena* and β-glucan upregulated the mRNA expression levels of IFN-γ, IL-6 and IL-10 and IL-12Rβ1 compared to the CCP group, although the difference was not significant (Figs. 4A, 4B, 4D, 4E). The IL-12 receptor (IL-12R) consists of IL-12Rβ1 and IL-12β2 [55-57]. The binding of IL-12 and IL-12R triggers the function of innate lymphocytes, such as Th1 and NK cells, through important instructive signaling and promotion of IFN-γ production [55, 58, 59]. IFN-γ is a representative cytokine secreted by T cells and plays an important role in recognizing and removing extracellular pathogens. The presence of IFN-γ maintains the activity of CD4+ and CD8+ T cells [60], activates the proliferation of Th1 cells, and plays critical roles in increasing Toll-like receptor expression, activating macrophages, and improving phagocytosis [61, 62]. Increased gene expression of IL-12Rβ1 may exert a partial enhancing effect on NK cell activity, and enhanced NK cell activity may lead to amplification of IFN-γ gene expression (Figs. 3, 4A, 4B). The expression of proinflammatory cytokines (TNF-α and IL-6) was increased because IFN-γ activated macrophages and monocytes (Figs. 4C, 4D). Similarly, Nakashima *et al*. reported *Euglena* and β-glucan intake increased secretion of cytokines such as IFN-γ, TNF-α, IL-12, IL-6 and IL-10 in vivo [10], and Qingqing *et al*. determined β-glucan significantly induced proinflammatory cytokines (TNF-α and IL-6) in RAW 264.7 macrophages [63]. Until now, no studies have been conducted on the effect of *Euglena* on cytokine expression in vivo models induced immunosuppression by CCP to the best of our knowledge. The secretion of IL-10, a representative anti-inflammatory cytokine, inhibits the proinflammatory reactions of CD4+ T cells. However, IL-10 promotes the apoptosis and memory abilities of CD8+ T cells, and promotes the survival, proliferation, and antibody production capabilities of B cells. Therefore, the anti-inflammatory cytokine IL-10 contributes to maintaining the homeostasis of the immune system by inhibiting the activation of inflammation-inducing mechanisms and enhancing adaptive immune responses and phagocytosis [64-69].

Dectin-1 is a C-type lectin receptor (CLR) that functions as a transmembrane pattern-recognition receptor (PRR) by binding a variety of β-1,3-linked and β-1,6-linked glucans, which is a polysaccharide on pathogens such as fungi and some bacteria [70]. Dectin-1 is mainly engaged in linking innate and adaptive immunity [71, 72]. The predominant expression of dectin-1 has been observed in inflammatory cells and immune cells activated upon pathogen entry [73], and dectin-1 has been predominantly found on DCs and macrophages [74-77]. In contrast to other PRRs, dectin-1 does not directly bind to substances derived from microorganisms; dectin-1 binds to microorganisms, playing a central role in innate immunity [78]. The binding between dectin-1 and β-glucans results in the activation and recruitment of spleen tyrosine kinase (Syk). As a result, NFAT- and NF-κB-induced transcription induces cytokine and chemokine production [6, 79-81]. Dectin-1 mRNA is widely expressed in several tissues, of which many are also expressed in monocytes of livers and a lungs [71, 82, 83].In the present study, the administration of 500 mg of *Euglena*/kg B.W. protected the mRNA expression of decin-1 against CCP treatment-induced inhibition in the lungs and livers (Figs. 5A, 5B). These results indicate that *Euglena* may enhance the innate immune system against pathogens, especially fungi. This present study is first time to determine that administration of *Euglena*, and β-1,3-glucan extracted from *Euglena* increased dectin-1 gene expression, so this study is expected to provide useful information for further studies on functionality of *Euglena*.

*Euglena* stores carbohydrates in the form of paramylon (β-glucan) throughout the cytoplasm in rod-like bodies [84, 85]. The bioactive function of paramylon has been previously revealed. The binding of β-1,3-glucan with dectin-1 activates the innate immune response, which leads to the production of reactive oxygen species (ROS) and inflammatory cytokines by activating nuclear factor kappa-light-chain-enhancer of activated B cells [15, 86]. Therefore, we expected that the stimulation of dectin-1 by *Euglena* administration would promote innate immune responses and that dectin-1 would induce inflammatory cytokine expression in lymphocytes.

In summary, *Euglena* enhanced dectin-1 expression rather than stimulating lymphocytes directly, and then enhanced the function of DCs and macrophages. These results suggest that *Euglena* improves the front-line defenses against external pathogen invasion by enhancing the innate immune system. It is expected that these findings will provide useful experimental evidence for further research on *E. gracilis* in vivo and in human studies. In conclusion, the present study demonstrated that *Euglena* may have the potential to stimulate the immune system by inducing the expression of dectin-1 which recognized the β-1,3- glucan in *E. gracilis*. Moreover, *Euglena* slightly increases NK cell activity and cytokine secretion by T cells and B cells.

However, the pathway mechanisms of cytokine secretion in splenocytes and subsequent reactions due to the expression of dectin-1 in macrophages or DCs were not demonstrated, which is a limitation of this study. Further studies are needed to reveal the mechanism of cytokine and dectin-1 action. Taken together, *Euglena gracilis* powder has the potential to be used as a worthy natural immunopotentiator in developing functional food products.

## Figures and Tables

**Table 1:** The composition of *Euglena gracilis* powder.

Components	Value
Energy (Kcal/100g)	293.21
Carbohydrate (%)	65.56
Crude Protein (%)	21.75
Crude Fat (%)	7.07
Moisture (%)	2.41
Ash (%)	3.21
β-glucan (mg/g)	665.61

**Table 2:** Primers used for qRT- PCR analysis.

Gene	Forward (5’-3’)	Reverse (5'-3')
IL-12Rβ1	ccccagcgctttagcttt	gccaatgtatccgagactgc
TNF-α	tcttctcattcctgcttgtgg	ggtctgggccatagaactga
IL-6	gctaccaaactggatataatcagga	ccaggtagctatggtactccagaa
IFN-γ	atctggaggaactggcaaaa	ttcaagacttcaaagagtctgagg
IL-10	cagagccacatgctcctaga	tgtccagctggtcctttgtt
Dectin-1	atggttctgggaggatggat	cctggggagctgtatttctg
GAPDH	aagagggatgctgcccttac	ccattttgtctacgggacga

IL-12Rβ1, interleukin 12 receptor beta 1; TNF-α, tumor necrosis factor-alpha; IL-6, interleukin-6; IFN-γ, interferon-gamma; IL-10, interleukin-10; dectin-1, C-type lectin domain family 7 member A; and GAPDH, glyceraldehyde 3-phosphate dehydrogenase.
